# *Acinetobacter*‐Induced Endocarditis Post Cupping Therapy: Case Report

**DOI:** 10.1002/ccr3.70490

**Published:** 2025-05-07

**Authors:** Fateh Kashkash, Sarya Swed, Ali Mustafa, Saleh Azizi, Agyad Bakkour, Ahmad Abdalla, Mohammad Alkammar, Wael Hafez

**Affiliations:** ^1^ Department of Pulmonology Aleppo University Hospital Aleppo Syria; ^2^ Faculty of Medicine Aleppo University Aleppo Syria; ^3^ Department of Internal Medicine Al‐Mouwasat University Hospital Damascus Syria; ^4^ Sheikh Khalifa Medical City Abu Dhabi UAE; ^5^ Royal College of Surgeons in Ireland (RCSI) Dublin Ireland; ^6^ Mediclinic Alnoor Hospital Abu Dhabi UAE; ^7^ NMC Royal Hospital Abu Dhabi UAE; ^8^ Medical Research and Clinical Studies Institute The National Research Centre Cairo Egypt

**Keywords:** *Acinetobacter*, cupping, endocarditis, pulmonary emboli, wet cupping

## Abstract

Acinetobacter‐induced endocarditis, though rare and typically associated with immunocompromised patients, can occur in healthy individuals. This case underscores the importance of considering uncommon pathogens and detailed patient histories, particularly following procedures such as cupping therapy, in the diagnosis of endocarditis.

## 1 Introduction

Endocarditis is a life‐threatening condition characterized by inflammation of the endocardium and inner lining of the heart, typically resulting from bacterial infection [1]. This condition often affects the heart valves and, if not promptly treated, can lead to severe complications such as heart failure, systemic embolism, and multi‐organ failure [1]. Right‐sided endocarditis predominantly affects individuals with intravenous drug use, implanted cardiac devices, and congenital heart defects. Common causative organisms include *Staphylococcus aureus* and *Streptococcus viridans* [2]. However, in recent years, less common pathogens, including *Acinetobacter* species, have gained recognition because of their resistance to multiple antibiotics and their association with healthcare‐associated infections [3].

*Acinetobacter* is an opportunistic pathogen that is frequently involved in nosocomial infections, particularly in immunocompromised patients or those with prolonged hospital stays [4]. Its clinical presentation can be subtle and easily mistaken for more common infections, leading to diagnostic delays and challenges in effective management.

In recent years, there has been a resurgence in the popularity of alternative and traditional medicine practices, such as cupping therapy, across various regions. Cupping therapy, an ancient practice rooted in several traditional healing systems, involves creating suction on the skin to promote blood flow and purportedly remove toxins [5]. Despite its widespread use, particularly in the Middle East and Asia, this procedure carries risks, particularly when performed under non‐sterile conditions or in sensitive body areas.

In this case report, we present a rare case of endocarditis caused by *Acinetobacter* species in a 17‐year‐old male who was diagnosed following cupping therapy performed in the inguinal region.

## 2 Case Presentation/Examination

A 17‐year‐old male presented with a 24‐h history of persistent high fever accompanied by persistent fatigue, diarrhea, and vomiting. Upon admission, his vital signs were as follows: blood pressure, 105/75 mmHg; respiratory rate, 25 breaths/min; and body temperature, 41°C. The patient had no significant past medical or chronic illnesses.

Several significant abnormalities were noted in initial laboratory investigations. The white blood cell count was 17,900/mm^3^, indicating significant leukocytosis. The platelet count was 60,000/mm^3^, indicative of thrombocytopenia. Additionally, the hemoglobin level was measured at 10.4 g/dL and the hematocrit at 30.2%, both of which are considered low. The erythrocyte sedimentation rate (ESR) was elevated, with values of 38 mm at 1 h and 79 mm at 2 h. Furthermore, uric acid levels were elevated at 10.9 mg/dL, while calcium levels were low at 7.98 mg/dL, indicating hypocalcemia. Lastly, the C‐reactive protein (CRP) level was significantly elevated at 221 mg/L, further reflecting the inflammatory state of the patient.

**FIGURE 1:**
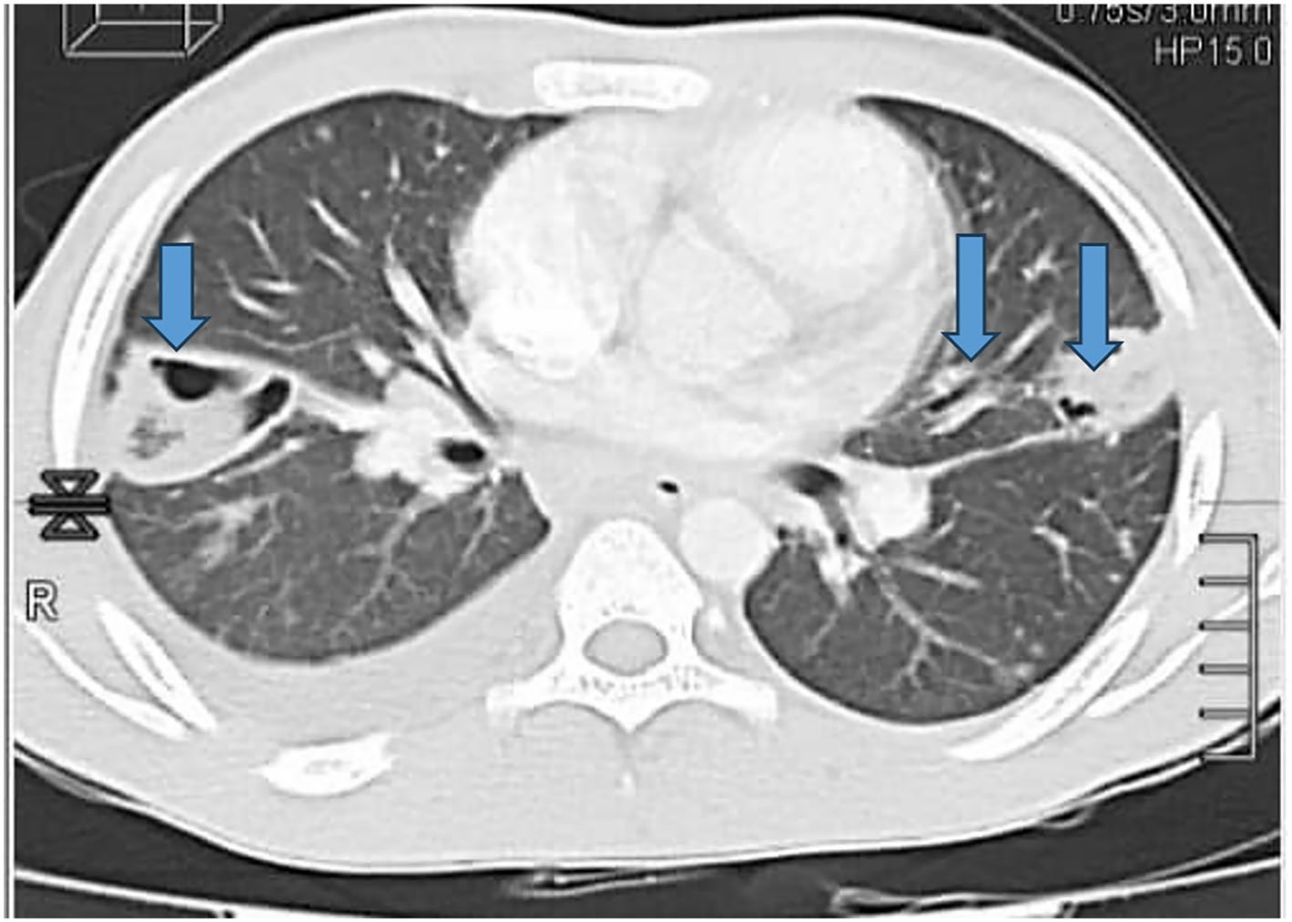
Chest CT scan showing punctate lesions consistent with septic emboli from the cardiac infection and associated lobar pneumonia (See arrows in blue).

Abdominal and pelvic ultrasound revealed marked hepatomegaly, with the liver measuring > 26 cm in diameter and splenomegaly measuring 15 cm. Additionally, a small amount of free fluid was detected in the abdominal cavity between intestinal loops. These findings suggest that the patient may have experienced systemic venous congestion as a consequence of tricuspid regurgitation. A chest CT scan revealed punctate lesions suggestive of septic emboli originating from the cardiac infection site, along with lobar pneumonia (Figure 1). Transesophageal echocardiography conducted on the same day showed vegetation around the native tricuspid valve (Video 1). No other potential sources of bacterial transmission were identified upon further investigation. The patient had no recent history of hospitalization, surgery, intravenous drug use, or exposure to individuals infected with Acinetobacter infections. Notably, 15 days before the onset of symptoms, the patient underwent cupping therapy in the inguinal region, performed by a traditional medicine practitioner. The procedure was performed outside a medical facility, raising concerns regarding environmental sterility.

**VIDEO 1:** Transesophageal echocardiography demonstrating vegetation around the native tricuspid valve. Video content can be viewed at https://onlinelibrary.wiley.com/doi/10.1002/ccr3.70490

## 3 Methods (Differential Diagnosis, Investigations, and Treatment)

Empirical antibiotic therapy with vancomycin, gentamicin, and piperacillin‐tazobactam was initiated, and blood cultures were obtained. Multiple samples were drawn from the peripheral and central veins to detect pathogens. The samples were inoculated into aerobic and anaerobic blood culture bottles and incubated at 35°C–37°C, and the results confirmed the presence of non‐motile, oxidase‐negative, catalase‐positive, strictly aerobic, gram‐negative *coccobacilli*. Automated identification using the Micronaut‐E system confirmed that *Acinetobacter* species were the causative pathogens.

Antimicrobial susceptibility testing conducted using the Kirby‐Bauer disc diffusion method revealed that the organism was extensively drug‐resistant (XDR), showing sensitivity only to imipenem, sulfamethoxazole/trimethoprim, and tetracycline. Based on this susceptibility pattern, the treatment regimen was adjusted to imipenem‐cilastatin 1 g three times daily, doxycycline 100 mg twice daily, and trimethoprim/sulfamethoxazole 800/160 mg twice daily.

## 4 Conclusion and Results (Outcome and Follow‐Up)

**FIGURE 2:**
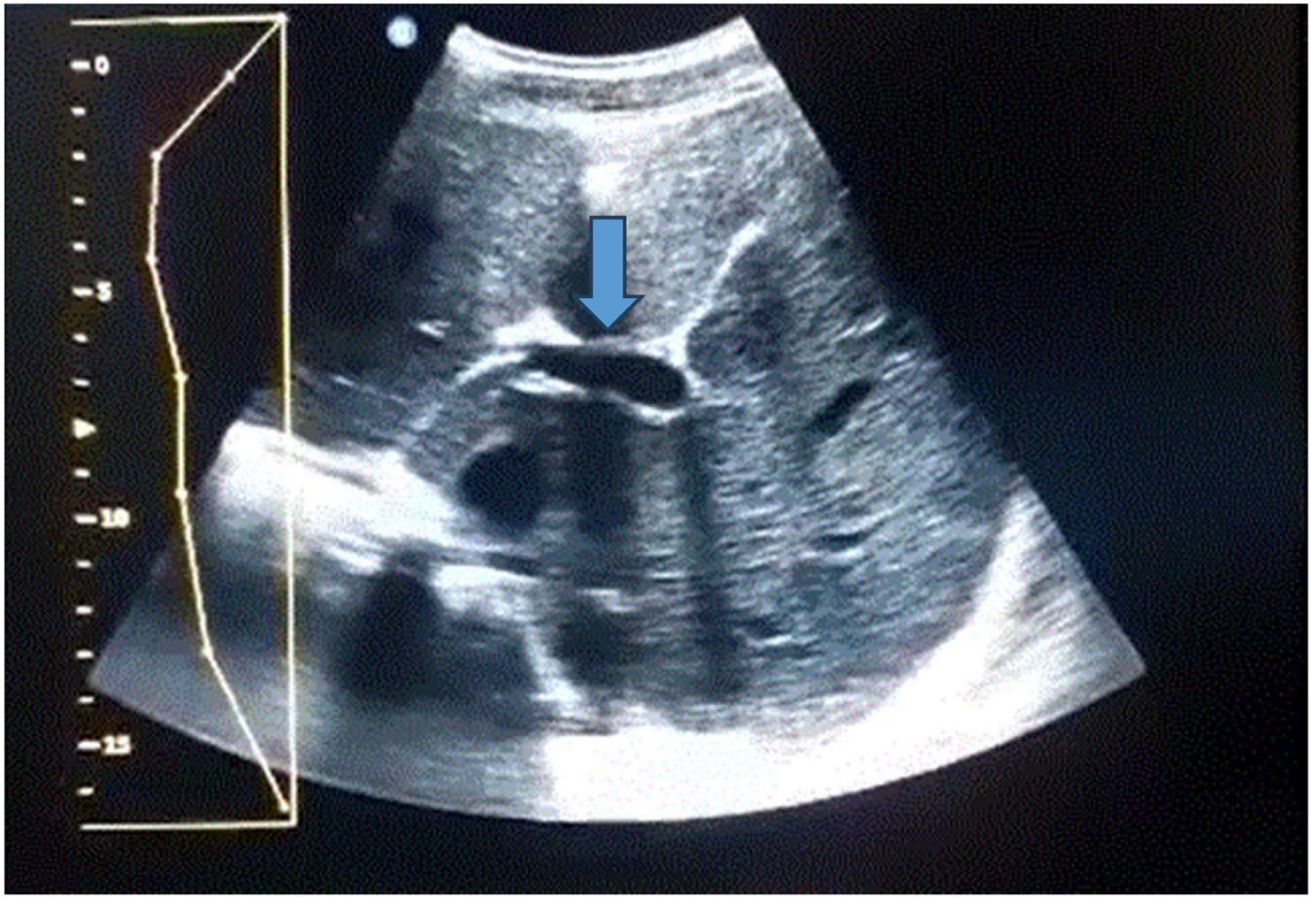
Abdominal ultrasound after 5 months of follow‐up.

After 14 days of follow‐up, the patient showed noticeable improvement in vital signs and fever. After 1 month of antibiotic treatment, the inflammatory panel was repeated, and the results were negative, indicating resolution of the infection. After 5 months of follow‐up, abdominal ultrasonography revealed only mild hepatic vein congestion and slight hepatomegaly (Figure 2).

## 5 Discussion

In this case, the patient developed endocarditis 15 days after undergoing cupping therapy in the inguinal region. The typical incubation period for Acinetobacter infections ranges from 4 to 40 days, aligning with the fifteen‐day interval between therapy and the onset of symptoms. This temporal relationship strengthens the likelihood that the procedure plays a role in the infection. Furthermore, cupping therapy, which often involves the creation of skin abrasions, may provide an entry point for bacteria. Given that no other potential sources of infection were identified, the cupping procedure, particularly if performed in a non‐sterile environment, raises significant concerns regarding its potential as a port of entry for Acinetobacter.

Typically, right‐sided endocarditis predominantly affects individuals with intravenous drug use (IVDU) and usually involves pathogens such as *Staphylococcus aureus* and *Pseudomonas aeruginosa* . For example, Shain et al. [6] reported a 25‐year‐old woman with a history of IVDU who developed right‐sided endocarditis due to methicillin‐resistant *Staphylococcus aureus* (MRSA). Her condition was marked by large tricuspid valve vegetation and septic pulmonary emboli. This typical presentation contrasts sharply with our case, where endocarditis was caused by *Acinetobacter* spp. following cupping therapy, which is a rare risk factor for this infection. This comparison underscores the typical pathogens and risk factors associated with right‐sided endocarditis, highlighting the unusual nature of this case.

Acinetobacter can often be found in moist areas of the skin, such as between the toes, groins, and armpits [7]. Recent data indicate that *Acinetobacter radioresistens* is an exceptionally rare pathogen responsible for bacteremia, with only a few documented cases [7, 8]. This bacterium is of particular concern because of its ability to develop resistance to carbapenems. The implications are serious, as *Acinetobacter* was identified as the causative agent of approximately 8500 infections among hospitalized patients and was linked to approximately 700 deaths in the United States in 2017 [9].

A systematic review of endocarditis caused by *Acinetobacter* species found that, among the native valves, the aortic valve was the most frequently affected, followed by the mitral valve [10].

Reports on endocarditis caused by *Acinetobacter baumannii* are rare. In the available literature, underlying heart disease is commonly cited as a risk factor, frequently observed in patients with prosthetic valves or pre‐existing heart valve disorders, as noted by Qian Chen in 2015 [11], Mauro Sturiale in 2016 [12]. In contrast, our case involves a 17‐year‐old previously healthy individual, which is highly atypical for *Acinetobacter*‐related endocarditis, highlighting the unusual nature of this infection in an otherwise healthy young patient.

Antibiotic therapy should be guided by antimicrobial susceptibility testing to ensure appropriateness. Delays in initiating correct treatment can negatively affect patient outcomes. In most documented cases of *Acinetobacter* endocarditis, the pathogen is multidrug‐resistant, and treatment decisions are based on the results of antibiotic susceptibility tests [7, 9].

This case highlights the potential risks associated with traditional medical practices and the importance of recognizing atypical pathogens in the etiology of endocarditis, particularly in patients without a history of cardiac disease. Early diagnosis and appropriate treatment are critical for preventing severe complications and improving patient outcomes.

## Author Contributions

**Fateh Kashkash:** writing – original draft. **Sarya Swed:** writing – original draft, writing – review and editing. **Ali Mustafa:** writing – review and editing. **Saleh Azizi:** writing – review and editing. **Agyad Bakkour:** writing – original draft, writing – review and editing. **Ahmad Abdalla:** writing – original draft, writing – review and editing. **Mohammad Alkammar:** writing – review and editing. **Wael Hafez:** supervision, validation, visualization, writing, review, and editing.

## Ethics Statement

Ethical approval was not required for this study in accordance with local or national guidelines.

## Consent

Written informed consent was obtained from the patient and the patient's family for publication of the case report and accompanying images.

## Conflicts of Interest

The authors declare no conflicts of interest.

## Data Availability

All data underlying the results are available as part of the article, and no additional source data are required.

## References

[1] V. Vilcant and O. Hai , “Bacterial Endocarditis,” in StatPearls (StatPearls Publishing, 2024), https://www.ncbi.nlm.nih.gov/books/NBK470547/. 29262218

[2] P. Brouqui and D. Raoult , “Endocarditis due to Rare and Fastidious Bacteria,” Clinical Microbiology Reviews 14, no. 1 (2001): 177–207, 10.1128/CMR.14.1.177-207.2001. 11148009 PMC88969

[3] L. C. Antunes , P. Visca , and K. J. Towner , “ *Acinetobacter baumannii*: Evolution of a Global Pathogen,” Pathogens and Disease 71 (2014): 292–301, 10.1111/2049-632X.12125. 24376225

[4] A. Y. Peleg , H. Seifert , and D. L. Paterson , “ *Acinetobacter baumannii* : Emergence of a Successful Pathogen,” Clinical Microbiology Reviews 21, no. 3 (2008): 538–582, 10.1128/CMR.00058-07. 18625687 PMC2493088

[5] J. I. Kim , M. S. Lee , D. H. Lee , K. Boddy , and E. Ernst , “Cupping for Treating Pain: A Systematic Review,” Evidence‐Based Complementary and Alternative Medicine 2011 (2011): 467014, 10.1093/ecam/nep035. 19423657 PMC3136528

[6] L. M. Shain , T. Ahmed , M. L. Bodine , and J. G. Bauman , “Drug Use‐Related Right‐Sided Infective Endocarditis Complicated by Empyema and Bronchopleural Fistula,” BMJ Case Reports 15, no. 1 (2022): e246663, 10.1136/bcr-2021-246663. PMC8762097 35027382

[7] M. F. Brady , Z. Jamal , and N. Pervin , “Acinetobacter,” in StatPearls (StatPearls Publishing, 2024), https://www.ncbi.nlm.nih.gov/books/NBK430784/. 28613535

[8] T. Wang , V. Costa , S. G. Jenkins , B. J. Hartman , and L. F. Westblade , “Acinetobacter Radioresistens Infection With Bacteremia and Pneumonia,” IDCases 15 (2019): e00495, 10.1016/j.idcr. 30906692 PMC6411504

[9] Centers for Disease Control and Prevention , “Carbapenem‐Resistant Acinetobacter,”, https://www.cdc.gov/drugresistance/pdf/threats‐report/acinetobacter‐508.pdf.

[10] P. Ioannou , V. Mavrikaki , and D. P. Kofteridis , “Infective Endocarditis by Acinetobacter Species: A Systematic Review,” Journal of Chemotherapy 33, no. 4 (2020): 203–215, 10.1080/1120009X.2020.1812804. 32875967

[11] Q. Chen , H. Cao , H. Lu , Z. H. Qiu , and J. J. He , “Bioprosthetic Tricuspid Valve Endocarditis Caused by *Acinetobacter baumannii* Complex, a Case Report and Brief Review of the Literature,” Journal of Cardiothoracic Surgery 10 (2015): 149. 26537904 10.1186/s13019-015-0377-8 PMC4634728

[12] M. Sturiale , C. Corpina , and L. Sturiale , “Endocarditis due to *Acinetobacter baumannii* ,” International Journal of Cardiology 209 (2016): 161–163. 26896613 10.1016/j.ijcard.2014.10.098

